# Editorial: The interactions between gastrointestinal microbiota and *Helicobacter pylori* in diseases

**DOI:** 10.3389/fcimb.2022.1043906

**Published:** 2022-09-27

**Authors:** Yi Hu, Yuan Zhuang, Hong-Yan Gou, Chuan Xie, Zhong-Ming Ge

**Affiliations:** ^1^ Department Of Gastroenterology, Digestive Disease Hospital, The First Affiliated Hospital of Nanchang University, Nanchang, China; ^2^ Department Of Gastroenterology, Shenzhen Hospital of The First Affiliated Hospital of Nanchang University, Shenzhen, China; ^3^ Department of Microbiology and Biochemical Pharmacy, College of Pharmacy, Third Military Medical University, Chongqing, China; ^4^ Shenzhen Research Institute, The Chinese University of Hong Kong, Shenzhen, China; ^5^ Division of Comparative Medicine, Massachusetts Institute of Technology, Cambridge, MA, United States

**Keywords:** *Helicobacter pylori*, eradication, gastric cancer, probiotic, microbiota

*Helicobacter pylori* (*H. pylori*) is a medically important pathogen colonizing the stomach, leading to the damage of gastric mucosa from chronic active gastritis, chronic atrophic gastritis, intestinal metaplasia and dysplasia to intestinal-type gastric cancer (GC) in a subset of infected subjects (Correa, 2013). *H. pylori* was defined as a carcinogen by the World Health Organization in 1994 and the U.S. Department of Health and Human Services in 2021. According to *Maastricht VI/Florence consensus report*, *H. pylori* infection is a primary cause for the development of GC, and *H. pylori* eradication prior to the stage of chronic atrophic gastritis is the most effective for the prevention of GC (Malfertheiner et al., 2022). It has been documented that the occurrence of spontaneous atrophic gastritis and gastrointestinal intraepithelial neoplasia (GIN) was not developed in germ-free (GF) transgenic insulin-gastrin (INS-GAS) mice by 13 months of age. However, *H. pylori* infection accelerated the development of GIN in GF INS-GAS mice, which was noted by 9 to 11 months post-*H. pylori* infection (equivalent to 11 to 13 months of mouse age). Comparing with *H. pylori*-infected INS-GAS mice with the complex gastric microbiota, *H. pylori* monoassociation caused less severe gastric lesions and delayed onset of GIN (Lofgren et al., 2011). In human patients, a distinct cluster of oral bacteria (*Peptostreptococcus* etc.) were associated with emergence and persistence of atrophy and intestinal metaplasia in subjects who developed atrophy 1 year after *H. pylori* eradication (Sung et al., 2020), which indicated that other organisms might be also involved in gastric inflammation and gastric carcinogenesis.

*H. pylori* infection was also shown to influence the composition, diversity and interactions of gastrointestinal microbiota. Bismuth-containing quadruple therapy was recommended as the first-line treatment of *H. pylori* infection in an era of increasing antibiotic resistance (Malfertheiner et al., 2022). The proton pump inhibitor, antibiotics and bismuth used in the regimens could induce the disruption of the microbiota in the short-term (Liou et al., 2019). However, the influence of *H. pylori* eradication on dysbiosis of microbiota in the long-term remained controversial depending on the different regimens (dual or triple or quadruple therapy). In this Research Topic, we aimed to focus on the influence of *H. pylori* infection or eradication on the gastrointestinal microbial community structure, as well as the role of the gastrointestinal microbiota or probiotic in *H. pylori*-related diseases. Totally, 7 articles (original research or review or meta-analysis) with 46 authors were included.

## *H. pylori* pathogenesis

Autophagy is a highly conserved catabolic process, which plays a double-edge role in cancer progression (Wang et al., 2021). Yang et al. presented an overview of autophagy in *H. pylori* infection and related GC with the focus on the relationship between *H. pylori* virulence factors and autophagy, core autophagy protein markers associated with *H. pylori* infection, the autophagy mediated signaling pathways involved in *H. pylori*-associated GC and potential drugs therapy targeting autophagy. This review provided the comprehensive knowledge of autophagy in *H. pylori* carcinogenesis and insights into the targeted autophagy therapies for treating *H. pylori*-associated GC. *H. pylori* acted as an initiating factor during the canonical pathological progression of GC by upregulating the inflammation of gastric mucosa. More recently, much attention has been drawn to the influence of *H. pylori* on the efficacy of tumor immunotherapy because emerging evidence shows the close associations between *H. pylori* infection and PD-1/PD-L1 blockade therapy (Shi et al., 2022). Wu et al. developed a scoring system based on three *H. pylori*-related core genes (*CRTAC1*, *BATF2*, and *CTHRC1*) for predicting the prognosis of GC and efficacy of anti-PD-1/L1 immunotherapy *via* the risk signature which was established and verified through comprehensive bioinformatic analyses of mutation patterns of GC samples from six cohorts and immunohistochemical experiments. This score system can help us better classify the phenotypes of GC, guide more personalized therapies and provide more accurate prediction of metastasis and prognosis of GC.

## The influence of *H. pylori* infection or eradication on the oral or gastrointestinal microbiota

*H. pylori* gastritis was defined as infectious disease by *Kyoto global consensus report*, and transmission routes of *H. pylori* infection include oral-oral, fecal-oral and gastro-oral (Sugano et al., 2015). The oral cavity has been considered as a potential reservoir of *H. pylori* infection and may be the source of the gastric microbiota (Schulz et al., 2018). In their review, Chen et al. summarized the changes and interactional mechanisms of *H. pylori* and oral microbiota: co-aggregation, endosymbiosis, and formation of symbiotic biofilm. Moreover, *H. pylori* infection could regulate the gut microbiota by changing pH of the environment, affecting the immune responses, differentially secreting bacterial virulence factors etc. *H. pylori* eradication could disrupt the gut microbiota to some extent mainly due to the use of drugs in the regimens. Xu et al. presented a review with emphasis on the effects of *H. pylori* infection on gastrointestinal microecology and diseases. The mechanisms include the regulation of gastrointestinal microecological environment, local pH value, cytokines and antimicrobial peptides, and immune responses. *H. pylori* is the dominant species of bacteria, accounting for more than 70% abundance of the gastric microbiota. *H. pylori* eradication could increase the alpha diversity indexes, decrease the microbial interactions, alter microbial community structure and microbial community structures in short-term and long-term follow-ups, which was supported by a systematic review and meta-analysis by Guo et al. It is still controversial regarding whether *H. pylori* eradication could restore gastric microbiota to uninfected status. Vonoprazan- amoxycillin (VA) dual therapy is a promising *H. pylori* regimen because of its high efficacy, safety and the avoidance of unnecessary antibiotics use (Hu and Lu, 2022). Hu et al. further demonstrated that the alteration of the gut microbiota induced by the VA dual therapy was restored at the confirmation time point (1 month after eradication), which indicated that the VA dual therapy has minimal negative effects on the gut microbiota. Meanwhile, the gut microbiota in *H. pylori*-infected individuals exhibit increased richness, diversity, and better evenness than *H. pylori*-negative patients.

## The role of probiotic in *H. pylori* eradication

Multiple studies have been conducted to explore the usefulness of probiotic in *H. pylori* eradication due to the decreased efficacy and increased side effects of *H. pylori* regimens and the alteration of gut microbiota induced by *H. pylori* regimens. The results remained inconclusive because of the differences in -probiotic strains, treatment duration, oral administration methods of probiotics, regimens and experimental subjects among different studies (Shi et al., 2019). Much attention is also paid to the role of probiotic in improving the eradication rate and decreasing the side effects as the first-line treatment of *H. pylori* infection. Interestingly, Qu et al. reported that *H. pylori*-infected patients with previous failure of *H. pylori* eradication then received *Saccharomyces boulardii* for 2 weeks before the rescue treatment. 28.0% of *H. pylori*-infected patients was confirmed to be negative for *H. pylori* after *Saccharomyces boulardii* treatment and avoided the rescue treatment, which provides a new insight into the use of probiotic in the treatment of *H. pylori* infection although additional investigations with a large sample size are needed for defining therapeutic potential of probiotics in *H. pylori* infection.

## Conclusion

In conclusion, we hope that the articles published in this Research Topic have represented the current understanding of the related fields and have provided new insights into *H. pylori* pathogenesis, the influence of *H. pylori* infection/eradication on the host gastrointestinal microbiota, and potential applications of probiotics in *H. pylori* treatment. Despite that there are limitations in these individual studies due to the lack of detailed mechanisms and relatively small sample sizes, we believe that the knowledge from these published original and review articles will be useful for guiding the future basic and clinical research in the field of *H. pylori* infection and microbiota.

## Author contributions

YH, YZ, HG, CX and ZG wrote and revised this article. All authors made a substantial, direct, and intellectual contribution to this work and approved it for publication.

## Funding

This study was supported by the National Natural Science Foundation of China (No. 82000531 and 82170580), the Project for Academic and Technical Leaders of Major Disciplines in Jiangxi Province (No. 20212BCJL23065), the Key Research and Development Program of Jiangxi Province (No. 20212BBG73018), the Youth Project of the Jiangxi Natural Science Foundation (No. 20202BABL216006).

## Acknowledgments

We thank all the editors, authors and reviewers who contribute with their relevant work to this Research Topic.

## Conflict of interest

The authors declare that the research was conducted in the absence of any commercial or financial relationships that could be construed as a potential conflict of interest.

## Publisher’s note

All claims expressed in this article are solely those of the authors and do not necessarily represent those of their affiliated organizations, or those of the publisher, the editors and the reviewers. Any product that may be evaluated in this article, or claim that may be made by its manufacturer, is not guaranteed or endorsed by the publisher.

## References

[B1] Correa P. (2013). Gastric cancer: Overview. Gastroenterol. Clin. North Am. 42 (2), 211–217. doi: 10.1016/j.gtc.2013.01.002 23639637 PMC3995345

[B2] Hu Y. Lu N. H. (2022). Letter: a promising helicobacter pylori regimen - vonoprazan-based therapy. Aliment Pharmacol. Ther. 56 (4), 752–753. doi: 10.1111/apt.17114 35879899

[B3] Liou J. M. Chen C. C. Chang C. M. Fang Y. J. Bair M. J. Chen P. Y. . (2019). Long-term changes of gut microbiota, antibiotic resistance, and metabolic parameters after helicobacter pylori eradication: A multicentre, open-label, randomised trial. Lancet Infect. Dis. 19 (10), 1109–1120. doi: 10.1016/S1473-3099(19)30272-5 31559966

[B4] Lofgren J. L. Whary M. T. Ge Z. Muthupalani S. Taylor N. S. Mobley M. . (2011). Lack of commensal flora in helicobacter pylori-infected INS-GAS mice reduces gastritis and delays intraepithelial neoplasia. Gastroenterology 140 (1), 210–220. doi: 10.1053/j.gastro.2010.09.048 20950613 PMC3006487

[B5] Malfertheiner P. Megraud F. Rokkas T. Gisbert J. P. Liou J. M. Schulz C. . (2022). Management of helicobacter pylori infection: The maastricht VI/Florence consensus report. Gut. doi: 10.1136/gutjnl-2022-327745

[B6] Schulz C. Schutte K. Koch N. Vilchez-Vargas R. Wos-Oxley M. L. Oxley A. . (2018). The active bacterial assemblages of the upper GI tract in individuals with and without helicobacter infection. Gut 67 (2), 216–225. doi: 10.1136/gutjnl-2016-312904 27920199

[B7] Shi X. Zhang J. Mo L. Shi J. Qin M. Huang X. (2019). Efficacy and safety of probiotics in eradicating helicobacter pylori: A network meta-analysis. Med. (Baltimore) 98 (15), e15180. doi: 10.1097/MD.0000000000015180 PMC6485819 30985706

[B8] Shi Y. Zheng H. Wang M. Ding S. (2022). Influence of helicobacter pylori infection on PD-1/PD-L1 blockade therapy needs more attention. Helicobacter 27 (2), e12878. doi: 10.1111/hel.12878 35112435

[B9] Sugano K. Tack J. Kuipers E. J. Graham D. Y. El-Omar E. M. Miura S. . (2015). Kyoto Global consensus report on helicobacter pylori gastritis. Gut 64 (9), 1353–1367. doi: 10.1136/gutjnl-2015-309252 26187502 PMC4552923

[B10] Sung J. Coker O. O. Chu E. Szeto C. H. Luk S. Lau H. . (2020). Gastric microbes associated with gastric inflammation, atrophy and intestinal metaplasia 1 year after helicobacter pylori eradication. Gut 69 (9), 1572–1580. doi: 10.1136/gutjnl-2019-319826 31974133 PMC7456733

[B11] Wang Y. Du J. Wu X. Abdelrehem A. Ren Y. Liu C. . (2021). Crosstalk between autophagy and microbiota in cancer progression. Mol. Cancer 20 (1), 163. doi: 10.1186/s12943-021-01461-0 34895252 PMC8665582

